# G_i_α proteins exhibit functional differences in the activation of ERK1/2, Akt and mTORC1 by growth factors in normal and breast cancer cells

**DOI:** 10.1186/1478-811X-12-10

**Published:** 2014-02-13

**Authors:** Zhanwei Wang, Rica Dela Cruz, Fang Ji, Sheng Guo, Jianhua Zhang, Ying Wang, Gen-Sheng Feng, Lutz Birnbaumer, Meisheng Jiang, Wen-Ming Chu

**Affiliations:** 1 Cancer Biology Program, University of Hawaii Cancer Center, 701 Ilalo Street, Honolulu, HI 96813, USA; 2 Department of Obstetrics and Gynecology, Ren Ji Hospital, Shanghai Jiao Tong University School of Medicine, Shanghai, P.R. China; 3 Department of Pediatrics, Shanghai 6th People’s Hospital, Shanghai Jiaotong University, Shanghai, P.R. China; 4 Department of Molecular and Medical Pharmacology, David Geffen School of Medicine at UCLA, Los Angeles, CA 90095, USA; 5 Department of Pathology, School of Medicine, University of California at San Diego, La Jolla, CA 92037, USA; 6 Laboratory of Neurobiology, Division of Intramural Research, National Institute of Environmental Health Sciences, National Institutes of Health, Research Triangle Park, NC 27709, USA

**Keywords:** G_i_α proteins, receptor tyrosine kinase (RTK), EGFR, Gab1, Shp2, EGF, Akt, mTORC1, ERK1/2, and breast cancer

## Abstract

**Background:**

In a classic model, G_i_α proteins including G_i1_α, G_i2_α and G_i3_α are important for transducing signals from G_i_α protein-coupled receptors (G_i_αPCRs) to their downstream cascades in response to hormones and neurotransmitters. Our previous study has suggested that G_i1_α, G_i2_α and G_i3_α are also important for the activation of the PI3K/Akt/mTORC1 pathway by epidermal growth factor (EGF) and its family members. However, a genetic role of these G_i_α proteins in the activation of extracellular signal-regulated protein kinase 1 and 2 (ERK1/2) by EGF is largely unknown. Further, it is not clear whether these G_i_α proteins are also engaged in the activation of both the Akt/mTORC1 and ERK1/2 pathways by other growth factor family members. Additionally, a role of these G_i_α proteins in breast cancer remains to be elucidated.

**Results:**

We found that Gi1/3 deficient MEFs with the low expression level of G_i2_α showed defective ERK1/2 activation by EGFs, IGF-1 and insulin, and Akt and mTORC1 activation by EGFs and FGFs. Gi1/2/3 knockdown breast cancer cells exhibited a similar defect in the activations and a defect in *in vitro* growth and invasion. The G_i_α proteins associated with RTKs, Gab1, FRS2 and Shp2 in breast cancer cells and their ablation impaired Gab1’s interactions with Shp2 in response to EGF and IGF-1, or with FRS2 and Grb2 in response to bFGF.

**Conclusions:**

G_i_α proteins differentially regulate the activation of Akt, mTORC1 and ERK1/2 by different families of growth factors. G_i_α proteins are important for breast cancer cell growth and invasion.

## Introduction

G_i_α proteins including G_i1_α, G_i2_α and G_i3_α transduce signals from G_i_α protein-coupled receptors (G_i_αPCRs) to downstream pathways in hormone and chemo-attractant signaling. Recent genetic evidence strongly suggests that G_i_α proteins also play an important role in cell growth and tumorigenesis. Active mutants of G_i2_α promote cell transformation and are associated with ovarian cancer [1,2], whereas G_i2_α deficiency results in the development of spontaneous colitis and colon cancer in mice [1]. Also, G_i3_α ablation impairs the antiautophagic response to insulin in mouse liver, and G_i2_α and G_i3_α double deficiency leads to severe embryonic growth retardation and the subsequent embryonic lethality [3], which, at least in part, are similar to phenotypes of many growth factor receptor deficiencies. However, how these G_i_α proteins are implicated in cell growth and tumorigenesis remains poorly understood.

It is known that the extracellular signal-regulated kinases (ERK1/2) and PI3K/Akt/mTORC1 pathways play an essential role in cell growth and malignant transformation. Both G_i_αPCRs [4] and growth factor receptors, which are also called receptor tyrosine kinases (RTKs), can activate them in response to their respective ligands. The G_i_αPCRs-triggered activation is mainly mediated by Gβγ, which can either directly bind and activate PI3K [5], or transmit signals to ERK1/2 through Src-, Shc- and Grb2- dependent and independent mechanisms [6-8]. This activation is sensitive to the treatment of *Bordetella pertussis* toxin (PTX), which blocks the interaction of G_i_α proteins with G_i_αPCRs [6,9,10].

The RTK-triggered activation of these two pathways by growth factors, including basic fibroblast growth factor (bFGF), epidermal growth factor (EGF), heparin-binding EGF-like growth factor (HB-EGF), insulin and insulin-like growth factor 1 (IGF-1) [11,12], is mainly mediated by adaptor proteins and the phosphatase Shp2. The established features of ERK1/2 activation by EGF, IGF-1 and insulin are that activated RTKs recruit Grb2, insulin receptor substrates (IRSs) and Grb2-associated binding protein 1 (Gab1) to interact with Shp2 leading to ERK1/2 activation [13-17]. Intriguingly, Gab1 is also recruited to the FGF receptor (FGFR) and forms a complex with Grb2 and the FGF receptor substrate 2 (FRS2), but it is not involved in ERK1/2 activation by FGFs [18]. Instead, FRS2 and Grb2 as well as their interaction are essential for ERK1/2 activation by FGFs. Further, Gab1 is not implicated in the activation of the PI3K/Akt/mTORC1 pathway by insulin or IGF-1. In its place, IRSs are recruited to insulin receptors (IRs) or IGF-1Rs, interact with PI3Kp85 and activate PI3K [19] generating PIP3 and triggering phosphorylation of Akt on threonine (T) 308 by PDK1 and serine (S) 473 by the mammalian target of rapamycin complex 2 (mTORC2). Activated Akt triggers the activation of the mammalian target of rapamycin complex 1 (mTORC1), which in turn phosphorylates 4E-BP1 and S6 kinase (S6K), which phosphorylates S6. Conversely, Gab1 is critical for EGFR- and FGFR-mediated activation of the PI3K/Akt/mTORC1 pathway by EGF or FGFs. Gab1 is tyrosine (Y)-phosphorylated and then interacts with Grb2 and PI3Kp85. These interactions are essential for PI3K activation by EGF and FGFs.

It is known that G_i_αPCRs can trans-activate RTKs, and that both of them share some downstream adaptor proteins such as Grb2 and Shc to activate ERK1/2. However, PTX and Gβ blocking peptides can inhibit the activation of Akt and ERK1/2 by G_i_αPCRs ligands, but not EGF [20,21]. Thus, it is unknown whether G_i_α proteins can regulate RTKs-mediated activation of the ERK1/2 and PI3K/Akt/mTORC1 pathways by grow factors.

We previously reported that loss of G_i1_α and G_i3_α in mouse embryonic fibroblasts (MEFs) resulted in a defect in Akt and mTORC1 activation by EGF [20]. Yet, it is largely unknown whether these G_i_α proteins regulate ERK1/2 activation by EGF or ERK1/2, Akt and mTORC1 activation by other growth factors. Additionally, a role of the G_i_α proteins in breast cancer pathogenesis is unrevealed. In this study, we investigated a role of the G_i_α proteins in ERK1/2, Akt and mTORC1 activation by different families of growth factors in mouse embryonic fibroblasts and in human breast cancer cells.

## Results

### G_i_α proteins differentially regulate the activation of the ERK1/2 and Akt/mTORC1 pathways by growth factors in mouse embryonic fibroblasts

To elucidate a role for G_i_α proteins in the activation of ERK1/2, Akt and mTORC1 in response to growth factors, we used wild type (WT) and G_i1_α and G_i3_α double knockout (Gi1/3 DKO) MEFs [20]. First, we examined the expression levels of G_i1_α, G_i2_α and G_i3_α in MEFs. As shown, both G_i2_α and G_i3_α were relatively abundant, but G_i1_α was expressed at a low level and it required a longer-exposure for a clear signal (Additional file 1: Figure S1A). Interestingly, G_i2_α expression level was obviously lower in Gi1/3DKO MEFs than that in WT MEFs (Additional file 1: Figure S1A) indicating that loss of G_i1_α and G_i3_α might subsequently influence the expression of G_i2_α in MEFs.

**Figure 1:**
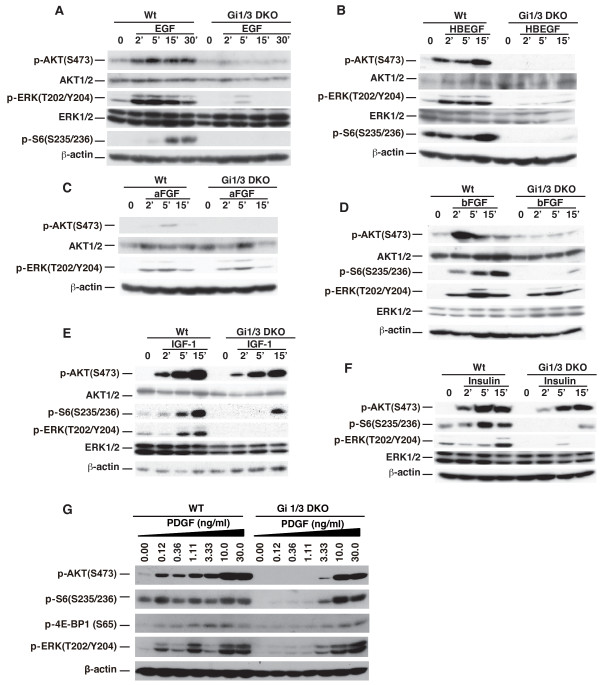
**G**_**i**_**α proteins regulate differential activation of Akt**, **mTORC1 and ERK1/2 by growth factors. (A-F)** WT and Gi1/3 DKO MEFs were treated with **(A)** EGF (100 ng/ml), **(B)** HB-EGF (100 ng/ml), **(C)** aFGF (25 ng/ml), **(D)** bFGF (25 ng/ml), **(E)** IGF-1 (20 ng/ml), and **(F)** insulin (1 μg/ml at the indicated time points. p-ERK (T202/Y204), p-AKT (S473), p-S6 (S235/236), or β-actin were detected by immunoblotting (IB) analysis. The experiments were performed more than six times and similar results were obtained. **(G)** WT and Gi1/3 DKO MEFs were treated with PDGF for 10 minutes (min) at the indicated doses. p-ERK(T202/Y204), p-AKT(S473), p-S6(S235/236), or β-actin were detected by IB. The experiment was repeated three times and similar results were obtained.

WT and Gi1/3 DKO MEFs were then treated with EGF, HB-EGF, aFGF, bFGF, IGF-1, insulin, and PDGF and the activation statuses of several intracellular signaling molecules were examined. The expression levels of major relay molecules in the ERK1/2 and Akt pathways in Gi1/3 DKO MEFs were similar to WT controls (Additional file 1: Figures S1B to S1D), whereas the phosphorylation levels of ERK1/2, Akt, and mTORC1’s downstream events including 4E-BP1 and S6 were altered (Figure 1). Kinetic experiments showed that Gi1/3 DKO MEFs exhibited a severe defect in phosphorylation of ERK1/2(T202/Y204), Akt (S473) and S6(S235/236) in response to EGF and HB-EGF (Figures 1A and 1B). Consistent with our previous study [20], loss of G_i2_α in MEFs did not have an apparent effect on ERK1/2 activation by EGF (Additional file 1: Figure S1E).

Surprisingly, loss of G_i1_α and G_i3_α largely impaired phosphorylation of Akt(S473) and S6(S235/236) in response to aFGF and bFGF, but had no apparent effect on ERK/12(T202/Y204) phosphorylation (Figures 1C and 1D). In contrast to the FGF response, the Gi1/3 deficiency largely decreased ERK1/2(T202/Y204) phosphorylation in response to IGF-1 and insulin (Figures 1E and 1F), whereas this deficiency only led to a minor defect in Akt(S473) phosphorylation [20]. Interestingly, S6(S235/236) phosphorylation was largely impaired in the deficient cells (Figures 1E and 1F).

To determine if G_i_αPCRs are involved in the activation of ERK1/2 by EGF, we treated cells with G_i_α protein inhibitor PTX. Consistent with our previous report, EGF-induced ERK1/2 activation was not reduced by PTX treatment (Additional file 1: Figure S1F) [20]. However, IGF-1-induced ERK1/2 activation was inhibited by PTX treatment (Additional file 1: Figure S1F).

Also, loss of G_i1_α and G_i3_α clearly reduced phosphorylation of Akt(S473), S6(S235/236), 4E-BP1(S65) and ERK1/2(T202/Y204) in response to PDGF at low doses of 0.12, 0.36 or 1.1 ng/ml (Figure 1G). However, the defect in this phosphorylation was not observed in the deficient cells in response to PDGF at high doses (Figure 1G) [20] indicating that G_i1_α and G_i3_α proteins regulate the activation of Akt, mTORC1 and ERK1/2 by PDGF in a dose dependent manner.

**Figure 2:**
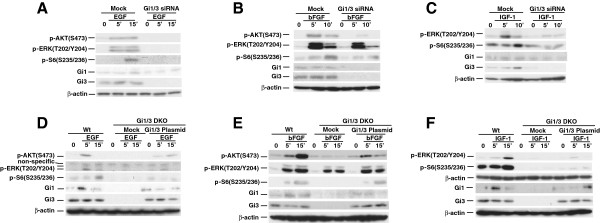
**Knockdown of G**_**i**_**α proteins down regulates Akt and ERK activation**, **and reintroduction of G**_**i**_**α proteins restores their activation by EGF, bFGF and IGF-1. (A-C)** WT MEFs transfected with siRNA duplexes specific for G_i1_α and G_i3_α were treated with **(A)** EGF (100 ng/ml), **(B)** bFGF (25 ng/ml), or **(C)** IGF-1 (20 ng/ml) for the indicated time points. p-AKT(S473), p-ERK(T202/Y204), p-S6(S235/236), β-actin, G_i1_α (Gi1) and G_i3_α (Gi3) were detected by IB. The experiments were repeated two to three times and similar results were obtained. **(D-F)** WT MEFs were transfected with empty vector and Gi1/3 DKO MEFs were transfected with WT G_i1_α, WT G_i3_α or empty expression vectors (5 μg). Cells were treated with **(D)** EGF (100 ng/ml), **(E)** bFGF (25 ng/ml) or **(F)** IGF-1 (20 ng/ml) for 5, 15 min, or left untreated. p-ERK(T202/Y204), p-AKT(S473), p-S6(S235/236), or β-actin, G_i1_α and G_i3_α were detected by IB. The experiments were repeated two to three times and similar results were obtained.

To confirm the above observations, we used RNA interference (RNAi) and reconstitution strategies. As shown, G_i3_α was clearly knocked down, whereas G_i1_α was weakly detected in MEFs (Figures 2A to 2C). Consistent with above results in Figure 1, the combined knockdown of Gi1/3 impaired ERK1/2(T202/Y204) phosphorylation in response to EGF and IGF-1, and reduced Akt(S473) and S6(S235/236) phosphorylation in response to EGF and bFGF (Figures 2A to 2C). Reintroduction of both G_i1_α and G_i3_α, or G_i3_α alone partially restored phosphorylation of ERK1/2(T202/Y204), or Akt(S473) and S6(S235/236) in Gi1/3 DKO MEFs in response to EGF, bFGF, or IGF-1 (Figures 2D to 2F).

Taken together, our experimental results demonstrate that G_i1_α, G_i2_α and G_i3_α are functionally distinct in RTKs-mediated activation of ERK1/2, Akt and mTORC1 in growth factor signaling (Table 1).

**Table 1:** Pathway activation by growth factors requires Ga proteins

	**EGF, HB- EGF, PDGF (low doses)**	**Insulin, IGF**	**aFGF, bFGF**	**PDGF (high doses)**
Akt-mTORC1 pathway	+	-	+	-
ERK pathway	+	+	-	-

+G_i_α proteins are required; - G_i_α proteins are not required.

### G_i_α proteins differentially regulate ERK1/2, Akt and mTORC1 activation in breast cancer cells and are important for their *in vitro* growth and invasion

**Figure 3:**
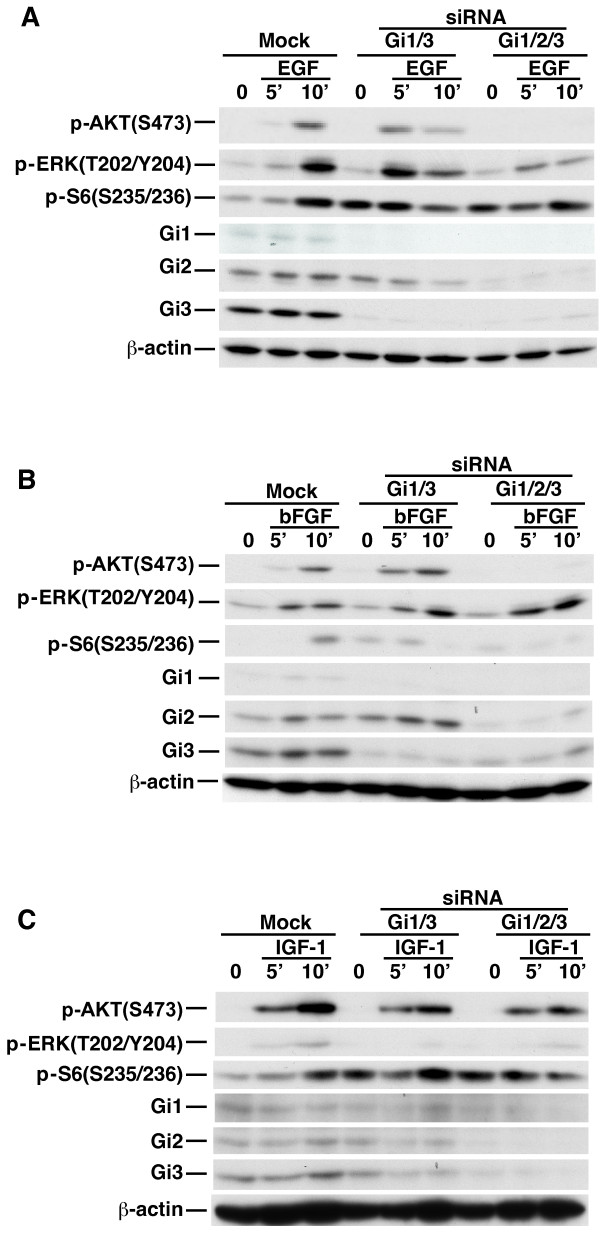
**G**_**i**_**α proteins regulate differential activation of ERK1/2, Akt and mTORC1 in breast cancer cells in response to EGF, bFGF and IGF-1.** MB231 cells were transfected with mock or siRNA duplexes (combination of G_i1_α and G_i3_α, or G_i1_α, G_i2_α and G_i3_α). Cells were then treated with **(A)** EGF (100 ng/ml), **(B)** bFGF (25 ng/ml), or **(C)** IGF-1 (20 ng/ml) for the indicated time durations. p-AKT (S473), p-ERK (T202/Y204), p-S6 (S235/236), G_i1_α (Gi1), G_i2_α (Gi2), G_i3_α (Gi3) or β-actin were detected by IB. The experiments were repeated two to three times and similar results were obtained.

Growth factor signaling plays a vital role in breast cancer pathogenesis [22-24]. We investigated whether G_i1_α, G_i2_α and G_3_α also regulate ERK1/2, Akt and mTORC1 activation by growth factors in breast cancer cells. Knockdown of Gi1/3 in MDA-MB-231-D3H2-LN (MB231) (Caliper Life Science), which expressed abundant G_i2_α and G_i3_α as well as detectable G_i1_α (Figures 3A to 3C), had a less striking effect on phosphorylation of ERK1/2(T202/Y204), Akt(S473) and S6(S235/236) than that in MEFs in response to EGF (Figure 3A). Knockdown of G_i2_α reduced Akt(S473) phosphorylation but did not impair phosphorylation of ERK1/2 and S6(S235/236) by EGF (Additional file 2: Figure S2). However, knockdown of G_i1_α, G_i2_α and G_3_α (Gi1/2/3) largely impaired this phosphorylation by EGF (Figure 3A) indicating that all three G_i_α proteins are important for the activation of Akt, mTORC1 and ERK1/2 by EGF. Similarly, knockdown of Gi1/2/3 led to a clear defect in phosphorylation of Akt(S473) and S6(S235/236) but not ERK1/2(T202/Y204) in response to bFGF (Figure 3B). Knockdown of either Gi1/3 or Gi1/2/3 clearly impaired ERK1/2 phosphorylation, and slightly reduced Akt(S473) phosphorylation in response to IGF-1 (Figure 3C).

**Figure 4:**
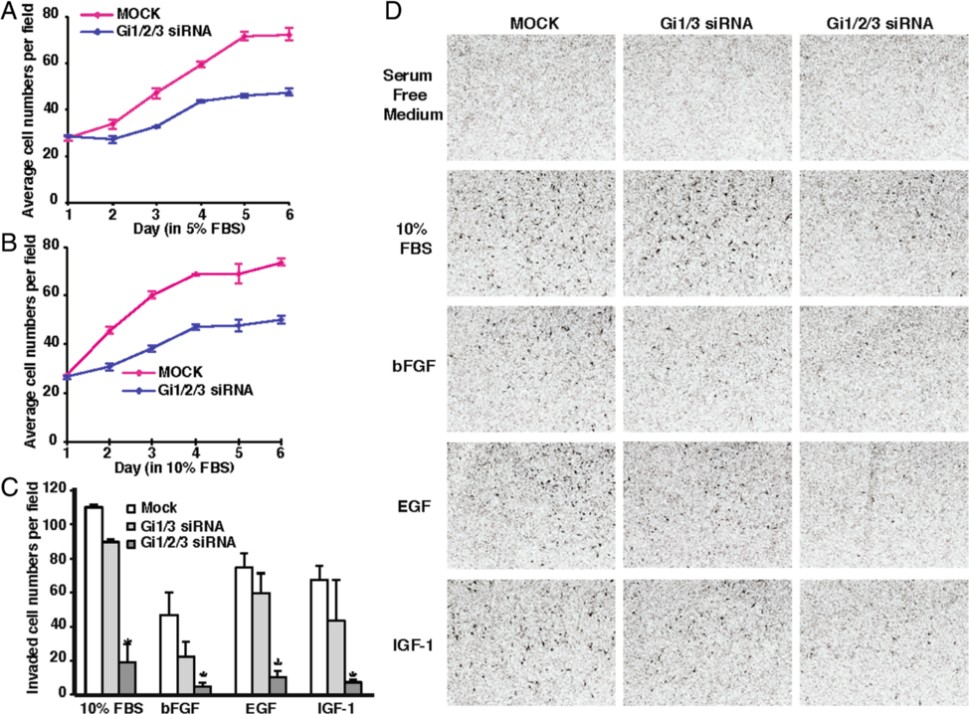
**G**_**i**_**α proteins are critical for breast cancer cell invasion in response to EGF, bFGF and IGF-1.** MB231 cells were transfected with scrambled or siRNA duplexes (combination of G_i1_α and G_i3_α, or G_i1_α, G_i2_α and G_i3_α). After 24 hours, cells were trypsinized. **(A-B)** Cells were seeded in 6-well plates, grown in DMEM containing 5% or 10% FBS and followed for up to 6 days. **(C-D)** Cells were seeded in new plates for another 48 hours. Cells were re-trypsinized, seeded into 8 μm transwells pre-coated with matrigel and exposed to EGF (100 ng/ml), bFGF (25 ng/ml), IGF-1 (20 ng/ml), 10% FBS or left untreated (with serum free medium) for 36 hours. Penetrated cells were stained and recorded by an Olympus CKX41 microscopy with an Infinity 2 camera. *P value <0.01 (invaded breast cancer cells transfected with G_i1_α, G_i2_α and G_i3_α siRNA duplexes were compared with cells transfected with scrambled siRNA duplexes). The experiments were repeated three times and similar results were obtained.

Because Akt and ERK1/2 signaling is required for cell growth and migration in response to growth factors [25], based on above observations, we reasoned that G_i_α proteins are involved in growth and invasion of breast cancers in response to EGF, bFGF, IGF-1 and FBS. To test this hypothesis we performed *in vitro* growth and invasion assays. We observed that knockdown of Gi1/2/3 not only clearly reduced the growth rate of breast cancer cells (Figures 4A and 4B, Additional file 3: Figure S3), but also significantly diminished their invasion ability in response to EGF, bFGF, IGF-1 and FBS (Figures 4C and 4D). Interestingly, knockdown of Gi1/3 reduced the invasion ability of breast cancer cells in response to these growth factors, although the reduction was not significant (Figures 4C and 4D). Additionally, knockdown of G_i2_α slightly reduced EGF-, bFGF-, or IGF-1-induced breast cancer invasion (Additional file 4: Figure S4). Taken together, our results suggest that G_i_α proteins promote growth and invasion of breast cancer cells.

**Figure 5:**
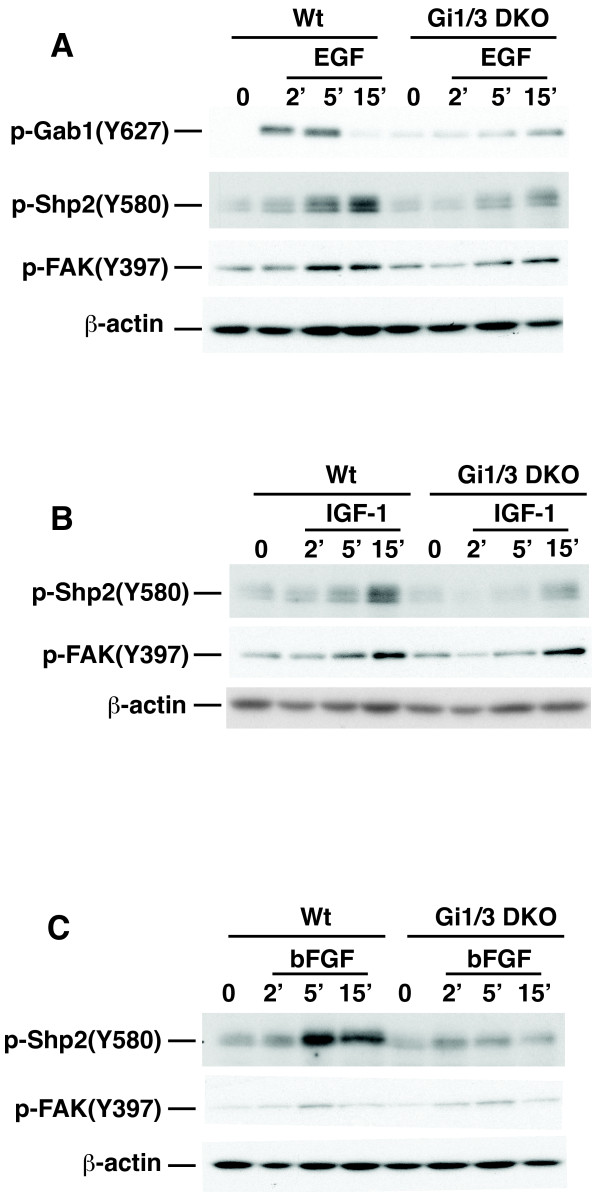
**G**_**i**_**α proteins are required for phosphorylation of Shp2 but not FAK1 in response to EGF, bFGF and IGF-1.** WT and Gi1/3 DKO MEFs were treated with **(A)** EGF (100 ng/ml), **(B)** bFGF (25 ng/ml), or **(C)** IGF-1 (20 ng/ml) for 0, 2, 5 or 15 min. p-FAK(Y397), p-Gab1(Y627), p-Shp2(Y508) and β-actin were detected by IB via anti-p-FAK(Y397), anti-p-Gab1(Y627), anti-p-Shp2(Y508) and anti-β-actin antibodies, respectively. Gab1 and Shp2 were also detected by IB (Additional file 5: Figure S5). Experiments were repeated two times and similar results were obtained.

To understand how G_i_α proteins regulate ERK1/2, Akt and mTORC1 activation and breast cancer invasion induced by growth factors in normal and breast cancer cells, we first examined whether G_i_α proteins are involved in the activation of FAK1, which is important for cell migration, and Shp2, which is critical for the activation of ERK1/2 by EGF and IGF-1. Consistent with our previous report [20] and above results, Gi1/3 DKO MEFs exhibited a defect in phosphorylation of Akt(S473), ERK1/2 and Gab1(Y627) in response to EGF (Figure 5A; Additional file 5: Figure S5A). Also, Gi1/3 DKO MEFs showed impaired S6(S235/236) and Shp2 phosphorylation in response to IGF-1 (Figures 5B; Additional file 5: Figure S5B), and diminished Shp2 phosphorylation in response to EGF (Figure 5A) and bFGF (Figure 5C), whereas they showed a slightly reduced FAK1(Y397) phosphorylation in response to EGF, bFGF and IGF-1 (Figures 5A to 5C).

**Figure 6:**
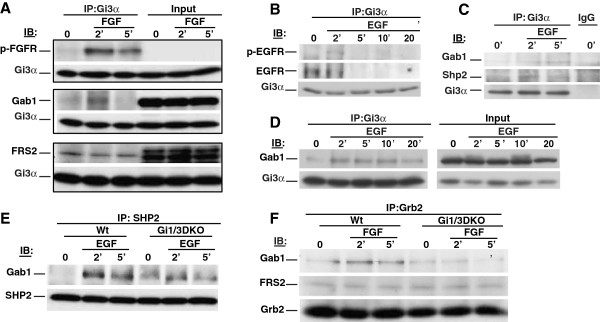
**G**_**i**_**α proteins associate with RTKs and their adaptor proteins in breast cancer cells**, **and are important for interactions of Gab1 with Shp2 or Grb2 in MEFs in response to EGF or bFGF. (A)** MB231 cells were treated with bFGF (25 ng/ml) for 0, 2 or 5 min. Whole cell lysates were prepared. The associations of G_i3_α with p-FGFR, Gab1 and FRS2 were determined by IB using anti-p-FGFR(Y766), anti-Gab1, anti-FRS2 and anti-G_i3_α antibodies, respectively. **(B-D)** MB231 cells were treated with EGF (100 ng/ml) for 0, 2, 5, 10 or 20 min. The associations of G_i3_α with p-EGFR, EGFR **(B)**, Gab1 and Shp2 **(C & D)** were determined by IB using anti-p-EGFR (Y1173), anti-EGFR, anti-Gab1, anti-Shp2 and anti-G_i3_α antibodies, respectively. **(E & F)** WT and Gi1/3 DKO MEFs were treated with EGF or bFGF for 0, 2 or 5 min. The association of Shp2 with Gab1 in response to EGF **(E)**, and the associations of Grb2 with Gab1 and FRS2 in response to bFGF **(F)** were determined by IB. Experiments were repeated two to four times. Similar results were obtained.

Next, we examined whether G_i_α proteins associate with growth factor receptors, their downstream adaptor proteins and Shp2. As shown, bFGF induced G_i3_α’s associations with p-FGFR and Gab1 (Figure 6A), but did not further augment the pre-association of G_i3_α with FRS2 (Figure 6A). EGF induced the associations of G_i3_α with p-EGFR, Gab1 and Shp2 (Figures 6B to 6D) in breast cancer cells.

Because Gab1’s association with Shp2 is critical for ERK1/2 activation by EGF and IGF-1 [15,16,26], and because Grb2’s association with FRS2 is critical for this activation by bFGF [18,27], we determined if G_i_α proteins are important for these associations. As shown, EGF induced Gab1’s interaction with Shp2 in WT cells, but this interaction was impaired in Gi1/3 DKO MEFs (Figure 6E). The interaction of Gab1 with Grb2, which is critical for activation of the PI3K/Akt pathway by FGF [18], was also impaired in Gi1/3/ DKO MEFs (Figure 6F). Conversely, Grb2’s interaction with FRS2 was intact in Gi1/3 DKO MEFs (Figure 6F). Taken together, our results suggest that G_i_α proteins are important for Gab1’s interaction with Shp2 in response to EGF and IGF-1, and Grb2’s interaction with Gab1 in response to bFGF.

## Discussion

G_i_α proteins are known to transduce signals from G_i_αPCRs to their downstream cascades and function at the inner surface of the cell membrane. Here, we demonstrate that G_i_α proteins also act as transducers for differential RTK-mediated growth factor signaling. G_i_α proteins form a complex with RTKs and are important for phosphorylation of Gab1 and Shp2, the association of Gab1 with Shp2, as well as the interaction of Gab1 with Grb2 leading to activation of the ERK1/2 and Akt/mTORC1 signaling pathways.

### Functional differences among G_i_α proteins in the regulation of growth factor signaling

Genetic evidence reveals that G_i2/3_α DKO mice die between embryonic days 9 and 10 due to severe mass growth retardation [3]. Although the exact death reason is currently unclear, the phenotype of the embryonic lethality strongly suggests that G_i_α proteins play an essential role in the cell proliferation and growth and survival of embryos.

There are several avenues for G_i_α proteins to contribute to proliferation and growth. One of them is the activation of G_i_αPCRs, which transduce signals from their agonists to their downstream PTX-sensitive signal cascades such as the PI3K/Akt/mTORC1 and ERK1/2 signaling pathways [4]. Activated G_i_αPCRs induce guanine nucleotide exchange at G_i_α proteins leading to their dissociation from Gβγ subunits. Dissociated Gβγ can trigger the activation of the both pathways [6-8].

Another route is the crosstalk between G_i_αPCRs and RTKs. RTKs can be transactivated by their agonists, which are either expressively induced by activation of G_i_αPCRs or secreted via G_i_αPCR-induced shedding of precursors of growth factors such as pro-HB-EGF [28,29]. Secreted HB-EGF binds to EGFR triggering the activation of multiple important signaling pathways including the PI3K/Akt/mTORC1 and ERK1/2 pathways. The G_i_αPCR-mediated shedding of HB-EGF is PTX sensitive, whereas EGFR-mediated activation of the PI3K/Akt/mTORC1 and ERK1/2 pathways by EGF is PTX insensitive [20,21]. In this study, we also found that PTX did not block the activation of ERK1/2 by EGF (Additional file 1: Figure S1F).

We demonstrate a possible third avenue in which G_i_α proteins differentially regulate RTK-mediated activation of the PI3K/Akt/mTORC1 and ERK1/2 signaling pathways. We previously showed that G_i_α proteins acted downstream of EGFR, but upstream of Gab1, leading to Gab1 phosphorylation and its subsequent interaction with PI3Kp85 in PI3K/Akt/mTORC1 signaling [20]. In this study, we show that G_i_α proteins are also important for ERK1/2 activation by EGF. G_i_α proteins formed a complex with the EGFR, its adaptor Gab1 and phosphatase Shp2 in response to EGF, and loss of G_i_α proteins impaired EGF-induced interaction of Gab1 with Shp2, which is crucial for the activation of ERK1/2 by EGF [30]. As a consequence, loss of G_i_α proteins led to a defect in the activation of Akt and ERK1/2 by EGF and HB-EGF. However, the relationship between G_i_α proteins and EGFR is currently unclear. A recent study suggested that EGF induced the formation of a G_i_α-GIV/Girdin-EGFR complex activating the PI3K-Akt pathway, and the interaction of G_i3_α with EGFR was dependent on the guanine exchange factor (GEF) motif in GIV [31]. GIV catalyzed the conversion of GDP-G_i3_α to GTP-G_i3_α, and its GEF motif was important for autophosphorylation of EGFR and its localization and degradation in response to EGF [31]. However, it is unknown whether the conversion of GDP-G_i3_α to GTP-G_i3_α by GIV regulates its downstream Gβγ subunits in EGF signaling.

Unlike EGFRs, insulin receptor (IR), IGF-1 receptor (IGF-1R) and FGF receptors (FGFRs) have their own adaptor proteins: FRS2 and IRSs, respectively. FRS2 is crucial for intracellular signaling in response to FGF [18,32] and its interaction with Grb2 is critical for ERK1/2 activation by bFGF [33]. We found that this association was intact in G_i_α deficient cells. This explains why ERK1/2 activation by aFGF and bFGF was observed in G_i_α deficient cells. Further, FRS2 interacts with Gab1 and is required for Gab1’s associations with Grb2 and PI3Kp85 as well as PI3K activation [27,34]. We identified that FRS2’s interaction with Gab1 or the interaction of Gab1 with Grb2 was impaired in G_i_α deficient cells. As a result, the activation of Akt and mTORC1 by aFGF and bFGF was diminished in G_i_α deficient cells revealing a new G_i_α/FRS2/Grb2/Gab1-PI3K cascade in FGF signaling.

IR and IGFR bind IRSs, which directly interact with PI3Kp85 leading to the activation of PI3K, Akt and mTORC1 [35]. Therefore, the activation of Akt and mTORC1 by IGF-1 and insulin was not largely reduced in G_i_α-deficient MEFs or was even enhanced in Gab1-deficient liver in response to insulin [36]. Conversely, the activation of ERK1/2 was not altered in IRS-1-deficient cells in response to IGF-1 [19], indicating that other proteins are required for ERK1/2 activation by IGF-1. Indeed, our data showed that loss of G_i_α proteins severely impaired ERK1/2 activation by IGF-1 and insulin. Intriguingly, although loss of G_i_α proteins exhibited a minor defect in Akt activation, the S6 phosphorylation was clearly impaired in Gi1/3-deficient MEFs (Figures 1E and 1F). The possible interpretation is that S6 can also be phosphorylated by other protein kinases such as p90RSK, which is a substrate of ERK1/2 and is able to phosphorylate S6 when mTORC1 activity is inhibited [37]. In addition, our data showed that PTX was able to reduce insulin-induced ERK1/2 activation (Additional 1: Figure S1F). This result suggests that G_i_αPCRs may be involved in the activation of ERK1/2 by insulin.

### G_i_α proteins in breast cancer

G_i_α proteins are highly expressed in some breast cancer cells, especially in metastatic breast cancer cells [38]. G_i_α proteins have been implicated in breast cancer invasion in response to SDF1, which is the only ligand for G_i_αPCR CXCR4. Upregulation of CXCR4 is vital for Her2-mediated metastasis of breast cancer [39,40]. Interestingly, the association of G_i_α proteins with Gβγ is considered a prerequisite for functional CXCR4 [38]. In the present study, we found that G_i_α proteins not only regulated SDF1-mediated invasion of breast cancer (data not shown), but also controlled EGF-, bFGF-, IGF-1- and serum-induced MB231 invasion. Additionally, G_i_α proteins regulated breast cancer growth.

Overexpression of RTKs such as EGFR family members and FGFRs is a hallmark of breast cancer. This phenotypic overexpression promotes activity of RTKs leading to overactivation of their downstream events and cascades, including Gabs [41,42], Shp2 [43] and the PI3K/Akt/mTORC1 and Ras/ERK1/2 signaling pathways leading to progression, invasion and metastasis of breast cancer [44,45]. We showed that G_i_α proteins associated with active EGFR and FGFR, as well as their downstream adaptor proteins and Shp2 in response to respective agonists. Further, G_i_α proteins can differentially regulate the activation of Akt, mTORC1 and ERK1/2 by EGF, bFGF and IGF-1 in breast cancer cells.

In summary, our results demonstrate that G_i_α proteins are important regulators of RTK signaling in normal cells and in breast cancer cells. G_i_α proteins differentially regulate the activation of Akt, mTORC1 and ERK1/2 by different growth factors. Given the fact that growth factors and their RTKs are critical for breast cancer progression, invasion and metastasis, pharmacological interference of G_i_α protein function may provide an alternative therapeutic strategy for breast cancer treatment.

## Materials and method

### Antibodies

Antibodies raised against EGFR (sc-03), G_i1_α (sc-391), G_i2_α (sc-7276), G_i3_α (sc-262), AKT1/2 (sc-8312), ERK1/2 (sc-94), FRS2 (sc-8318) and SH-PTP2 (Shp2, sc-280) were purchased from Santa Cruz Biotechnology (Santa Cruz, CA). Anti-β-actin antibody was purchased from Sigma-Aldrich (St. Louis, Mo). Anti-EGFR, anti-FGFR, anti-Gab1, anti-IGF-1R, and other anti-phospho antibodies were purchased from Cell Signaling Technology (Danvers, MA).

### Cell line and culture

WT and Gi1/3 DKO MEFs were characterized and cultured as previous described [20]. MDA-MB-231-D3H2-LN (MB231) cells were purchased from Caliper Life Science (Hopkinton, MA). Cells were cultured in DMEM high-glucose medium supplemented with 10%FBS in a CO_2_ incubator at 37°C.

### Immunoblotting analysis

WT, DKO, transfected or siRNA knockdown cells were starved overnight (o/n), and then treated with bFGF, EGF, HB-EGF (Invitrogen), aFGF (Cell Signaling), IGF-1 (Invitrogen and R & D Systems), or insulin (BD Pharmingen) for the indicated time points. Cells were washed twice with cold 1× phosphate buffered saline (PBS) and lysed with a lysis buffer [1% Triton ×-100, 150 mM NaCl, 50 mM Tris-Cl, pH 7.4, 0.5 mM EDTA, 1 mM sodium orthovanadate, 5 mM beta glycerol phosphate, 1× protease inhibitor cocktail (Roche, Indianapolis, IN)] [46]. Total of 30–60 μg protein lysates were boiled in the sample buffer, separated on 10% SDS-PAGE and transferred onto PVDF membranes (Millipore, Billerica, MA) followed by incubation with antibodies and detection with ECL (Pierce, Rockford, IL).

### Immunoprecipitation

Cells were cultured to 70% confluence and then starved o/n. Cells were treated with EGF, or bFGF for different time points and then lysed with the lysis buffer. For each sample, 600–800 μg of proteins were pre-cleared by incubation with 40 μl of protein A/G Sepharose (beads) (Amersham, IL) for 30 minutes at 4°C. After an overnight incubation at 4°C with specific antibodies (2–4 μg/mg protein), 40 μl of protein A/G bead suspensions were added to the complexes and incubated for another 2 hours. Then the beads were washed three times with washing buffer (lysis buffer containing 1 mM PMSF) and once with 1× PBS. The boiled proteins were separated on 10% SDS-PAGE gels and transferred onto PVDF membranes followed by incubation with different antibodies and detection with ECL.

### Plasmid transfection

Lipofectamine 2000 (Invitrogen) was used as the transfection reagent. 3.5×10^5^ Gi1/3 DKO cells were seeded in 6-well plates and cultured in DMEM supplemented with 10% FBS without antibiotics. Five μg G_i1_α or G_i3_α plasmids in 250 μl of serum free medium (SFM) and 10 μl Lipofectamine 2000 in 250 μl of SFM were gently mixed and incubated for 30 min. The plasmid-Lipofectamine complexes were then added to each well. After 5 hours the medium was replaced with complete medium. Cells were cultured for additional 14 hours, starved o/n and then treated with growth factors as described above.

### siRNA transfaction

Human and mouse G_i1_α, G_i2_α, and G_i3_α siRNA duplexes were purchased from Santa Cruz Biotechnology. Cells were cultured in 10% FBS DMEM in the absence of antibiotics to 70% confluence at the time of transfection. Lipofectimine RNAiMAX Reagent (7.5 μl, Invitrogen) in 250 μl SFM, and 3.5 μl of G_i1_α, G_i2_α or G_i3_α siRNA duplexes in another 250 μl SFM were gently combined, mixed, and incubated for 25 min at room temperature. The siRNA duplexes-RNAiMAX complexes were added to culture with a final RNA concentration of 10 nM in a total volume of 3 ml. After 16 hours, the medium was replaced by complete medium. Cells were cultured for another 24 hours and then starved o/n followed by treatment for immunoblotting analysis or *in vitro* invasion assay.

### Breast cancer cell *in vitro* invasion assay

Cell invasion assay was performed using Costar Transwell permeable support (8.0-μm pore size, Corning, NY) in 24-well plates. The starved mock, Gi1/3, Gi1/2/3 siRNA knockdown MB231 cells were harvested with trypsin, resuspended at 5×10^4^ cells/ml in SFM containing 0.1% BSA, and 200 μl of each type of cells were added to the upper chambers coated with Matrigel (1 mg/ml, BD Pharmingen). The lower chambers were filled with 600 μl SFM or SFM containing EGF, IGF-1, bFGF or 10%FBS. After 36 hours culture, cells were stained with HEME 3 Solution (Fisher Diagnostics, Middletown, VA), and non-invaded cells on the top of the transwell were removed with cotton swabs. Invaded cells were counted under an Olympus CKX41 microscopy (Olympus, Japan) and recorded by an Infinity 2 camera (Electron Microscopy Sciences, Hatfield, PA).

### Breast cancer cell growth assay

siRNA knockdown MB231 cells (scrambled siRNA duplexes (mock) or Gi1/2/3 siRNA duplexes) (1×10^4^ cells/well) were seeded in 6-well plates in DMEM medium containing 5% or 10% FBS. From day 1, at least three fields in each well were randomly selected for recording images and counting cells under an Olympus CKX41 microscopy with an Infinity 2 camera. Experiments were repeated twice.

### Statistical analysis

Statistical difference between two groups was evaluated using Student’s t test. *P* values less than 0.05 were considered significant. Data were presented as mean ± SD.

## Abbreviations

Giα: Inhibitory guanosine nucleotide-binding protein alpha; GiαPCRs: G_i_α protein-coupled receptors; EGF: Epidermal growth factor; HB-EGF: Heparin-binding EGF-like growth factor; FGF: Fibroblast growth factor; IGF-1: Insulin-like growth factor 1; PDGF: Platelet-derived growth factor; EGFR: EGF receptor; FGFR: FGF receptor; IR: Insulin receptor; IGF-1R: IGF-1 receptor; IRS: Insulin receptor substrate; FRS2: FGF receptor substrate 2; RTK: Receptor tyrosine kinase; Gab1: Grb2-associated-binding protein 1; Grb2: Growth factor receptor-bound protein 2; Shp2: SH2-containing tyrosine-specific protein phosphatase; ERK1/2: Extracellular signal-regulated protein kinase 1 and 2; PI3K: Phosphatidylinositide 3-kinase; mTORC1: Mammalian target of rapamycin complex 1; MEFs: Mouse embryonic fibroblasts; RNAi: RNA interference; siRNA: Small interfering RNA; KO: Knockout; PTX: *Bordetella pertussis* toxin.

## Competing interests

Authors declare that they do not have competing interests.

## Authors’ contributions

WMC and MJ designed experiments. WMC, MJ and ZW wrote the paper. LB and GSF edited and revised the paper, and also created key reagents. ZW, RDZ, FJ, SG, JZ, and YW did experiments. All authors read and approved the final manuscript.

## Supplementary Material

Additional file 1: Figure S1 (A) Left panel: Expression of G_i1_α (Gi1), G_i2_α (Gi2) and G_i3_α (Gi3) in WT and Gi1/3 DKO MEFs. WT and Gi1/3DKO MEFs were lysed and the expression levels of G_i_α proteins were determined by IB using anti-G_i1_α, anti-G_i2_α and anti-G_i3_α antibodies, respectively. Right panel: Expression of Gi1 and Gi2 in mouse brain tissues. Whole cell lysates of WT, G_i2_α^F/F^/Mx1^Cre^ (Gi2^F/F^/Mx1CRE) (without induction) and G_i2_α^F/F^/Gi1/3DKO (Gi2^F/F^/Gi1/3DKO) mouse brain tissues were used to determine the expression levels of Gi1 and Gi2. (B-D) The expression levels of Akt, ERK1/2, Gab1, EGFR, FGFR, IGF-1R and β-actin in WT and Gi1/3 DKO MEFs were determined by IB. (E) Loss of G_i2_α (Gi2) did not decrease ERK1/2 activation by EGF. (F) PTX did not block ERK activation by EGF, but reduced ERK1/2 activation by insulin. Click here for file

Additional file 2: Figure S2 Knockdown of G_i2_α reduced Akt(S473) phosphorylation, but not did inhibit ERK1/2 and S6 phosphorylation in response to EGF. MB231 cells were transfected with scrambled (mock) or G_i2_αsiRNA (Gi2siRNA) duplexes. Cells were treated with EGF for the indicated time points. p-Akt(S473S), p-ERK1/2(T202/Y204), p-S6(S235/236), G_i2_α (Gi2) and β-actin were detected by IB. Click here for file

Additional file 3: Figure S3 Knockdown of G_i1_α, G_i2_α and G_i3_α in breast cancer cells impairs their growth ability. MB231 cells were transfected with scrambled or siRNA duplexes [combination of G_i1_α, G_i2_α and G_i3_α (Gi1/2/3)]. After 24 hours, cells were trypsinized, seeded in 6-well plates and cultured in DMEM containing 5% or 10% FBS for up to 6 days. Cells were observed under an Olympus CKX41 microscope and recorded by an Infinity 2 camera. Click here for file

Additional file 4: Figure S4 Knockdown of G_i2_α in breast cancer cells did not impair their invasion ability in response to EGF, bFGF and IGF-1. MB231 cells were transfected with scrambled or G_i2_αsiRNA (Gi2siRNA) duplexes. Cells were seeded in new plates for another 48 hours. Cells were re-trypsinized, seeded into 8 μm transwells pre-coated with matrigel and exposed to EGF (100 ng/ml), bFGF (25 ng/ml), IGF-1 (20 ng/ml), or left untreated (with serum free medium) for 36 hours. Penetrated cells were stained and recorded. Click here for file

Additional file 5: Figure S5 Loss of G_i1_α and G_i3_α impairs Akt(S473) and ERK1/2(T202/Y204) phosphorylation in response to EGF, and S6(S235/236) phosphorylation in response to IGF-1. Click here for file
