# Supplementary figures and images for: Giα proteins exhibit functional differences in the activation of ERK1/2, Akt and mTORC1 by growth factors in normal and breast cancer cells
Source: Cell Commun Signal. 2014 Feb 13;12:10. doi: 10.1186/1478-811X-12-10 (PMC3937014; doi:10.1186/1478-811X-12-10)

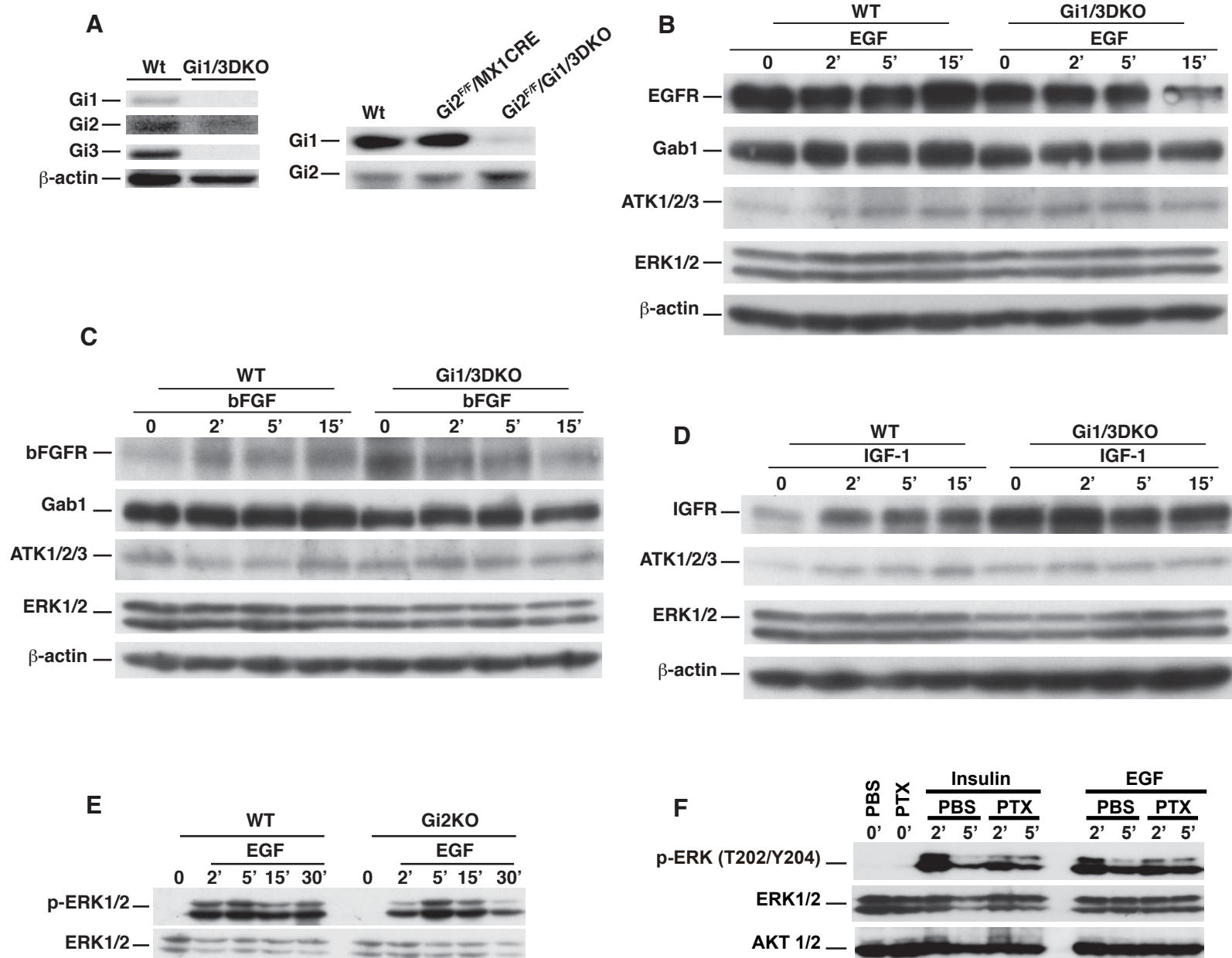

Supplement: Additional file 1: Figure S1 — (A) Left panel: Expression of Gi1α (Gi1), Gi2α (Gi2) and Gi3α (Gi3) in WT and Gi1/3 DKO MEFs. WT and Gi1/3DKO MEFs were lysed and the expression levels of Giα proteins were determined by IB using anti-Gi1α, anti-Gi2α and anti-Gi3α antibodies, respectively. Right panel: Expression of Gi1 and Gi2 in mouse brain tissues. Whole cell lysates of WT, Gi2αF/F/Mx1Cre (Gi2F/F/Mx1CRE) (without induction) and Gi2αF/F/Gi1/3DKO (Gi2F/F/Gi1/3DKO) mouse brain tissues were used to determine the expression levels of Gi1 and Gi2. (B-D) The expression levels of Akt, ERK1/2, Gab1, EGFR, FGFR, IGF-1R and β-actin in WT and Gi1/3 DKO MEFs were determined by IB. (E) Loss of Gi2α (Gi2) did not decrease ERK1/2 activation by EGF. (F) PTX did not block ERK activation by EGF, but reduced ERK1/2 activation by insulin. [file 1478-811X-12-10-S1.pdf]

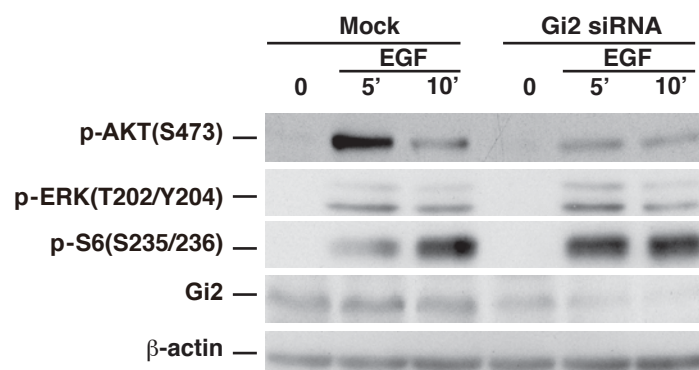

Supplement: Additional file 2: Figure S2 — Knockdown of Gi2α reduced Akt(S473) phosphorylation, but not did inhibit ERK1/2 and S6 phosphorylation in response to EGF. MB231 cells were transfected with scrambled (mock) or Gi2αsiRNA (Gi2siRNA) duplexes. Cells were treated with EGF for the indicated time points. p-Akt(S473S), p-ERK1/2(T202/Y204), p-S6(S235/236), Gi2α (Gi2) and β-actin were detected by IB. [file 1478-811X-12-10-S2.pdf]

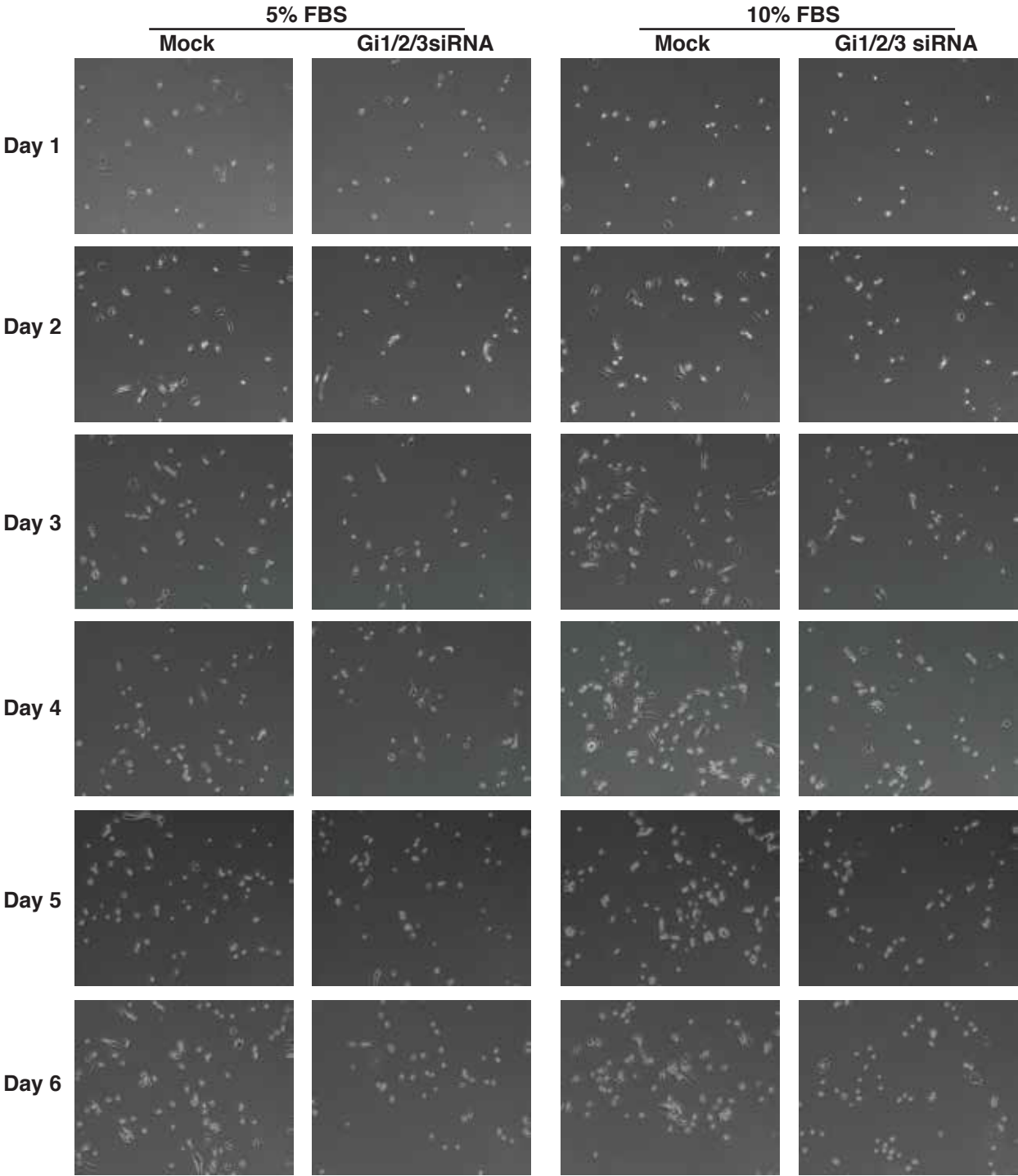

Supplement: Additional file 3: Figure S3 — Knockdown of Gi1α, Gi2α and Gi3α in breast cancer cells impairs their growth ability. MB231 cells were transfected with scrambled or siRNA duplexes [combination of Gi1α, Gi2α and Gi3α (Gi1/2/3)]. After 24 hours, cells were trypsinized, seeded in 6-well plates and cultured in DMEM containing 5% or 10% FBS for up to 6 days. Cells were observed under an Olympus CKX41 microscope and recorded by an Infinity 2 camera. [file 1478-811X-12-10-S3.pdf]

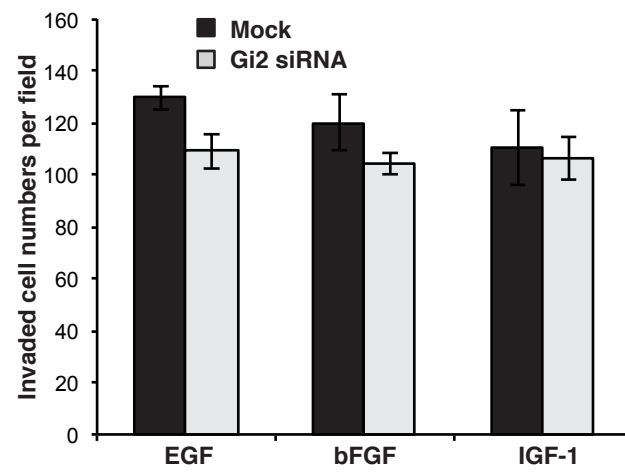

Supplement: Additional file 4: Figure S4 — Knockdown of Gi2α in breast cancer cells did not impair their invasion ability in response to EGF, bFGF and IGF-1. MB231 cells were transfected with scrambled or Gi2αsiRNA (Gi2siRNA) duplexes. Cells were seeded in new plates for another 48 hours. Cells were re-trypsinized, seeded into 8 μm transwells pre-coated with matrigel and exposed to EGF (100 ng/ml), bFGF (25 ng/ml), IGF-1 (20 ng/ml), or left untreated (with serum free medium) for 36 hours. Penetrated cells were stained and recorded. [file 1478-811X-12-10-S4.pdf]

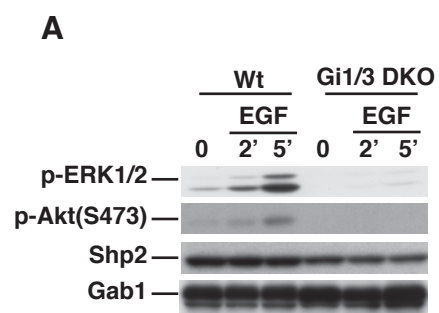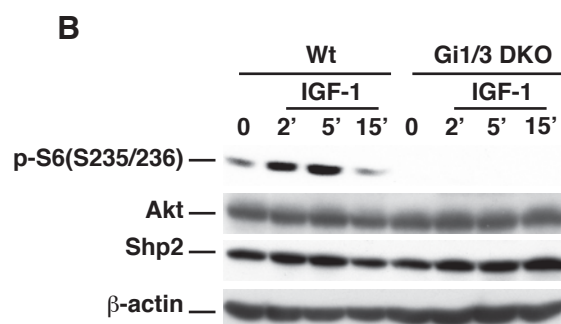

Supplement: Additional file 5: Figure S5 — Loss of Gi1α and Gi3α impairs Akt(S473) and ERK1/2(T202/Y204) phosphorylation in response to EGF, and S6(S235/236) phosphorylation in response to IGF-1. [file 1478-811X-12-10-S5.pdf]
